# Supplementary material for: BF2-Azadipyrromethene Fluorophores for Intraoperative Vital Structure Identification
Source: Molecules. 2023 Feb 25;28(5):2167. doi: 10.3390/molecules28052167 (PMC10004488; doi:10.3390/molecules28052167)
Supplement: Supplementary file 1 [file molecules-28-02167-s001.zip › molecules-2247313-supplementary.pdf]

Supporting Information

BF_2-Azadipyrromethene Fluorophores for Intraoperative Vital Structure Identification

Table of Contents

Synthesis S3

Figure S1. Alternate synthetic route to **2** S3

Synthetic procedure for compound **2** S3

Figure S2. Structures of **3a** and **5a** S4

Photophysical Data S4

Figure S3. Normalized absorbance and emission of **7-11** S4

In vivo imaging S6

Figure S4-S7. *In vivo* imaging of rodent model S6

Table S1-S3. Rodent toxicity study S8

Figure S8. *In vivo* ureter imaging of porcine model S8

NMR and mass spec data for compounds **2, 3, 3a, 4, 7-12** (Figure S9–Figure S18) S10

HPLC traces for compounds **3, 3a, 4, 7-12** (Figure S19) S28

References S30

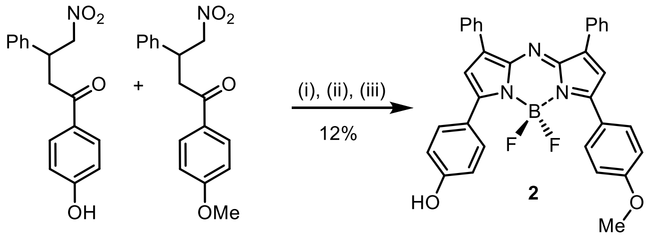

Figure S1. Alternate synthetic route to **2**. (i) NH_4OAc, EtOH, reflux, 16 h; (ii) $BF_3.OEt_2$, DIPEA, CH_2Cl_2 (anhydrous), rt, 16 h, (iii) silica gel purification, 12% over 2 steps.

Synthesis of BF_2 chelate of (*Z*)-4-(2-(((5-methoxyphenyl)-3-phenyl-1*H*-pyrrol-2-yl)imino)-3-phenyl-2*H*-pyrrol-2*H*-pyrrol-5-yl)phenol (2)

1-(4-Hydroxyphenyl)-4-nitro-3-phenylbutan-1-one[1] (1.50 g, 5.26 mmol) and 1-(4-methoxyphenyl)-4-nitro-3-phenylbutan-1-one[2] (1.57 g, 5.26 mmol) were dissolved in EtOH (37 mL) and ammonium acetate (15.0 g, 194.50 mmol) was added. The reaction was heated for 16 h under reflux with stirring. The reaction was cooled to rt and was further cooled to 0 °C in an ice bath. The resulting precipitate was vacuum filtered, washed with cold EtOH (3 x 20 mL), sat. $NaHCO_3$ (3 x 20 mL) and H_2O (3 x 20 mL), and vacuum dried overnight to give a crude mixture of azadipyrromethene products (1.24 g) as a blue-black solid. The crude mixture (1.22 g, ~2.46 mmol) was dissolved in dry CH_2Cl_2 (120 mL) and diisopropylethylamine (4.29 mL, 24.62 mmol, 10.0 eq.) was added under N_2. BF_3-diethyletherate (4.25 mL, 34.47 mmol, 14.0 eq.) was added dropwise and the reaction stirred at rt for 16 h. The solvent was removed by rotary evaporation, the crude residue dissolved in EtOAc (120 mL) and washed with sat. $NaHCO_3$ (2 x 120 mL), H_2O (2 x 120 mL) and brine (2 x 120 mL). The combined organic layers were dried over Na_2SO_4, filtered and concentrated by rotary evaporation yielding a crude residue. The crude was dry loaded (CH_2Cl_2 and silica) onto a silica column and purified by silica gel chromatography eluting with a gradient of CH_2Cl_2 (100 %) going to CH_2Cl_2 / EtOAc (9:1) yielding the product **2** as a red metallic solid (340.1 g, 12 %), mp 145-147 °C. ^{1}H NMR (400 MHz, DMSO-d^6) δ: 10.57 (s, 1H), 8.19 – 8.10 (m, 8H), 7.64 (s, 1H), 7.59 – 7.43 (m, 7H), 7.15 (d, *J* = 8.5 Hz, 2H), 6.96 (d, *J* = 8.4 Hz, 2H), 3.89 (s, 3H) ppm; ^{13}C NMR (101 MHz, DMSO-d^6) δ: 161.7, 161.4, 158.5, 156.1, 144.9, 144.0, 142.3, 141.2, 132.4, 132.0, 131.8, 131.6, 129.6, 129.3, 129.1, 129.0, 128.7, 123.5, 121.4, 120.1, 119.2, 116.0, 114.4, 55.6 ppm; ^{19}F NMR (376 MHz, DMSO-d^6) δ: –130.44 (q, 32.5 Hz) ppm. HRMS (ES): m/z calcd for $C_{33}H_{23}BF_2N_3O_2$ [M-H]$^-$ 542.1863; found 542.1864.

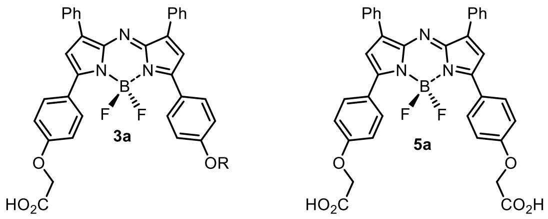

Figure S2. Structures of mono and bis-carboxylic acid functionalized fluorophores **3a** and **5a** employed in the synthesis of mono- and bis- activated ester fluorochromes **4** and **6**.

A

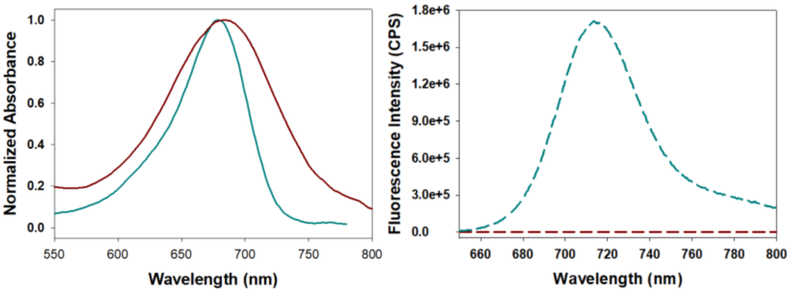

B

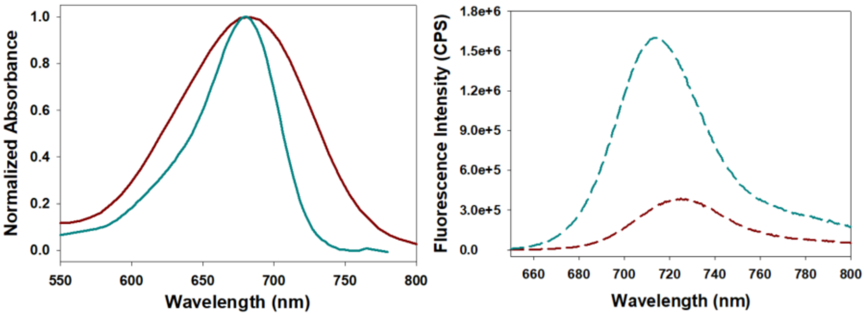

C

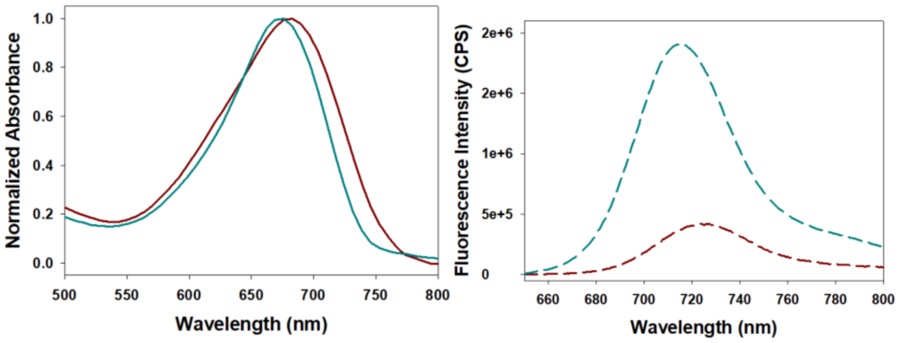

D

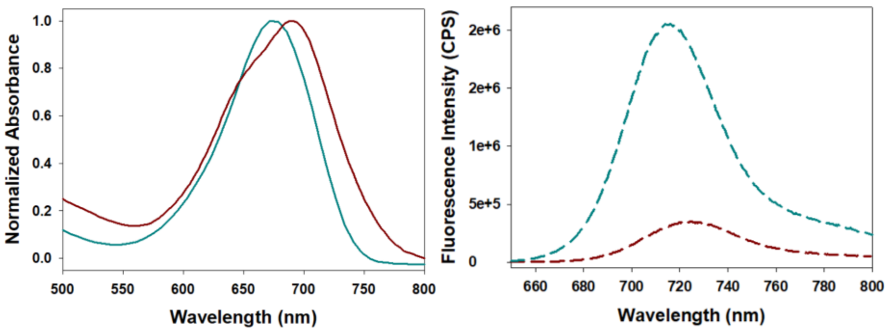

E

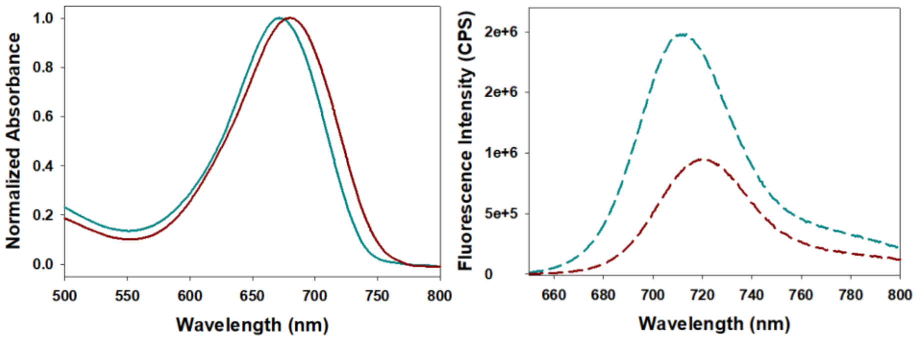

Figure S3. Absorbance (left, normalized) and emission (right) spectra in CH_3CN (cyan) and H_2O (red) of (A) **7** ; (B) **8** ; (C) **9** ; D **10** ; (E) **11**. Concentration 2 μM with 2.5 nm slit widths used for fluorescence spectra.

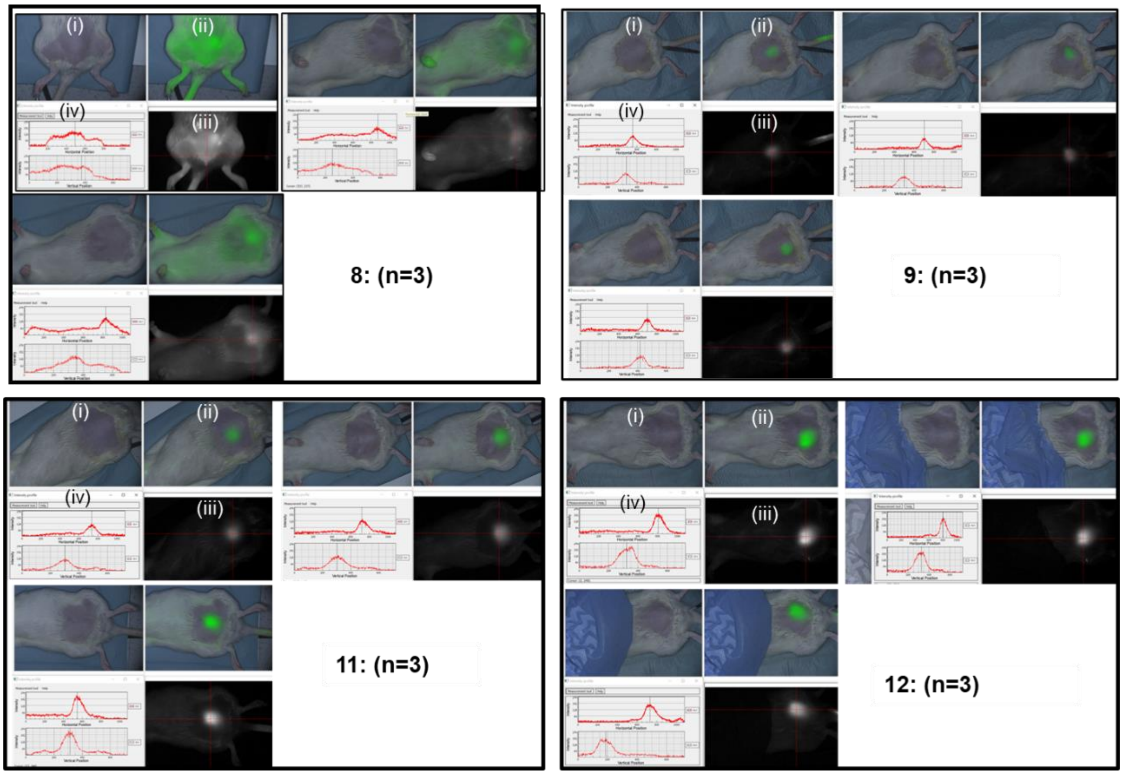

Figure S4. Representative images of the preliminary screening of fluorophores **8**, **9**, **11** and **12** (n=3, 1 mg/Kg). Fluorescence intensity from the bladder measured 60 min post administration (i) white light image; (ii) fluorescence (green) overlaid on white light image; (iii) fluorescence shown as black and white for clarity; (iv) averaged pixel analysis of fluorescence intensity through the bladder region of interest for **8** (128 au), **9** (98 au), **11** (155 au), **12** (186 au).

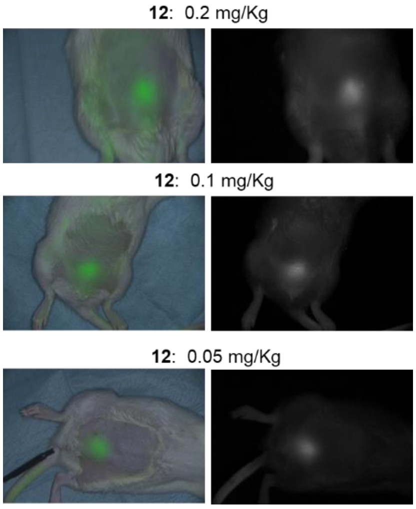

Figure S5. Representative images showing fluorescence from bladder for dose de-escalation study in rodents for **12**.

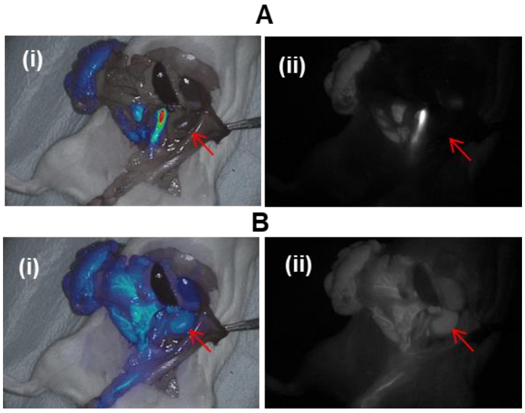

Figure S6. Fluorescence observed immediately upon tail vein administration of **12**. (A) at injection (B) 40 sec later. (i) fluorescence overlaid on white light image; (ii) fluorescence shown as black and white for clarity. Red arrow indicates a kidney.

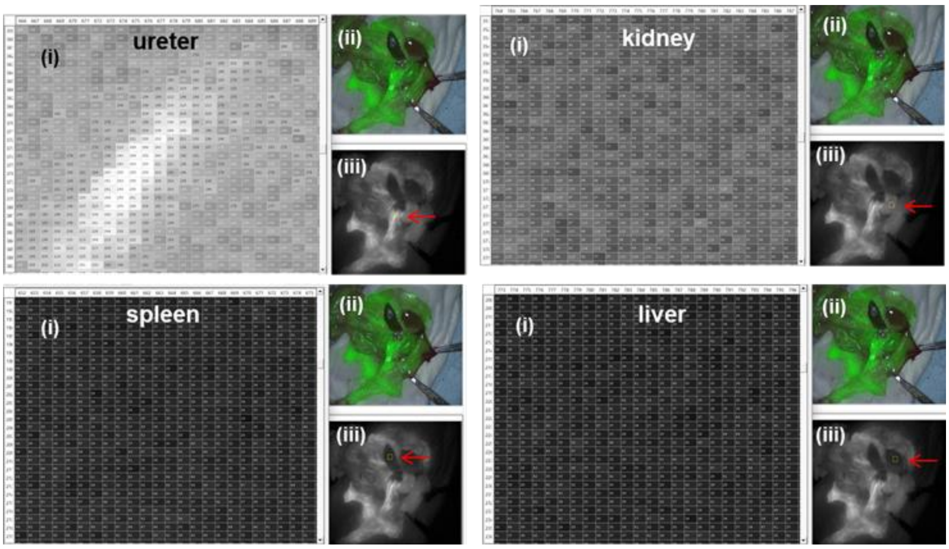

Figure S7. Representative pixel intensity analysis of four regions of interest taken 50 min post administration of **12**. Measured ureter (averaged intensity 167.7 au), kidney (99.3 au), spleen (35.1 au), liver (38.3 au) intensity values. (i) Pixel fluorescence intensity values for measured FOV in yellow box. (ii) fluorescence (green) overlaid on white light image; (iii) fluorescence shown as black and white for clarity. Red arrows indicates yellow box areas from which intensity values were recorded.

Table S1. Rodent urine study confirming renal excretion of **12**. Percentage collection of **12** is calculated from mmol of **12** injected versus mmol of **12** collected 90 min post injection.

entry	mg/kg of **12** injected	mmol of **12** injected	mmol of **12** in urine sample [a]	% of **12** collected
1	2.0	3.77×10^{-5}	1.44×10^{-5}	30
2	0.2	3.77×10^{-6}	1.24×10^{-6}	33
3	0.1	1.88×10^{-6}	5.66×10^{-7}	30
4	0.05	9.42×10^{-7}	2.4×10^{-7}	26

[a] Calculated using measured urine volume and standard abs / emission curves of **12**.

Table S2. Percentage body weight contributions of brain, spleen kidneys and liver of male Sprague-Dawley rats on day 8 following total dose intravenous administrations of **12** at 14 (2 x 7 daily administrations) and 10 mg/kg for each dose versus control, n = 3.

**Group**	**Dosage (mg/Kg)**	**Brain (%)**	**Spleen (%)**	**Kidney (%)**	**Liver (%)**
Control (PSB)	0	0.77	0.21	0.86	3.17
**12**	14 (2 x 7)	0.73	0.27	0.87	3.36
**12**	10	0.69	0.24	0.95	3.38

Table S3. Selected blood chemistry data for male Sprague-Dawley rats following intravenous administration of **12** at 14 mg/Kg (2 mg/Kg per day for 7 days) and a single dose at 10 mg/kg, n = 3.

**Group**	**AST IU/L**	**ALT IU/L**	**Creatinine µmol/L**	**Urea mmol/L**	**Glucose mmol/L**
Control (vehicle)	79 (17.1)	50 (7.9)	26.3 (2.3)	4.4 (0.2)	6.49 (0.6)
**12** (14 mg/Kg)	69 (3.5)	43 (1.2)	26.0 (1.2)	5.5 (0.4)	6.3 (0.8)
**12** (10 mg/Kg)	69 (0.6)	46 (3.6)	25.0 (1.3)	4.6 (0.9)	6.42 (0.7)

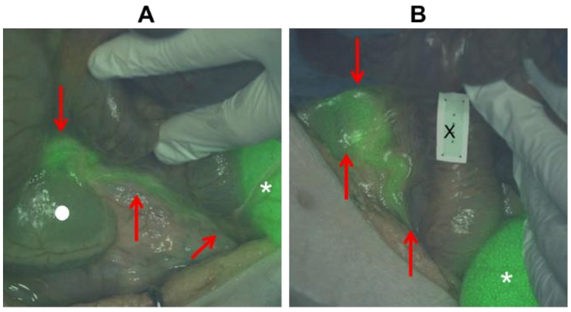

Figure S8. Additional representative images showing ureter identification in porcine model following treatment with **12** (0.5 mg/kg). (A) 25 min post administration (B) 120 min post administration. Red arrows indicate ureter, circle indicates kidney.

Figure S9: Analytical spectra for **2.**

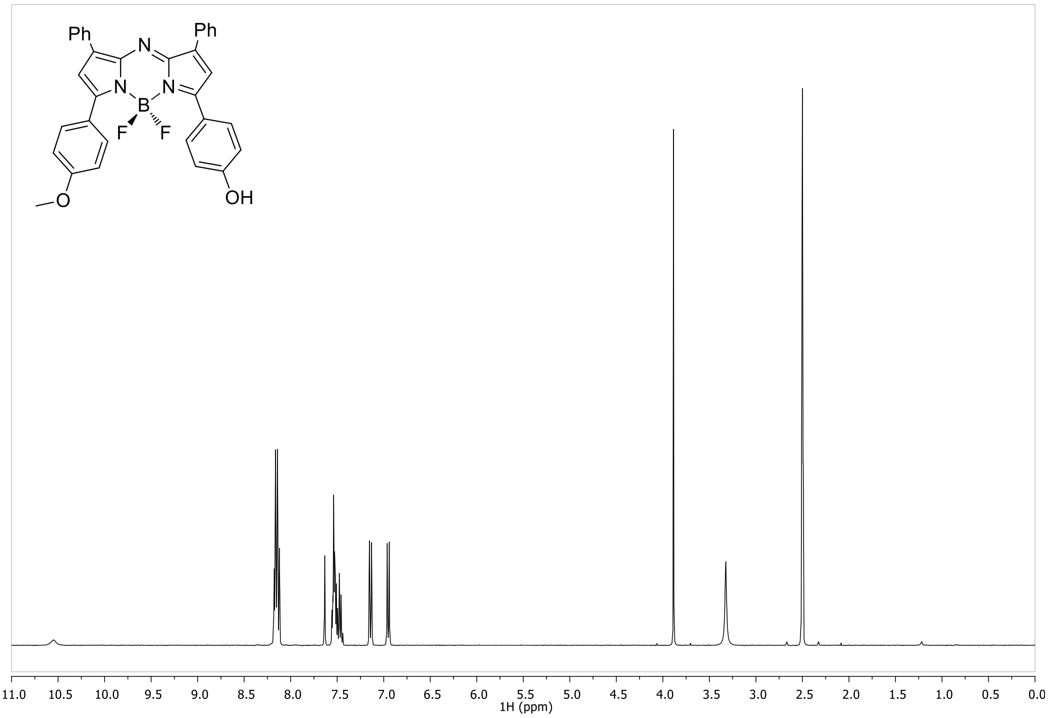

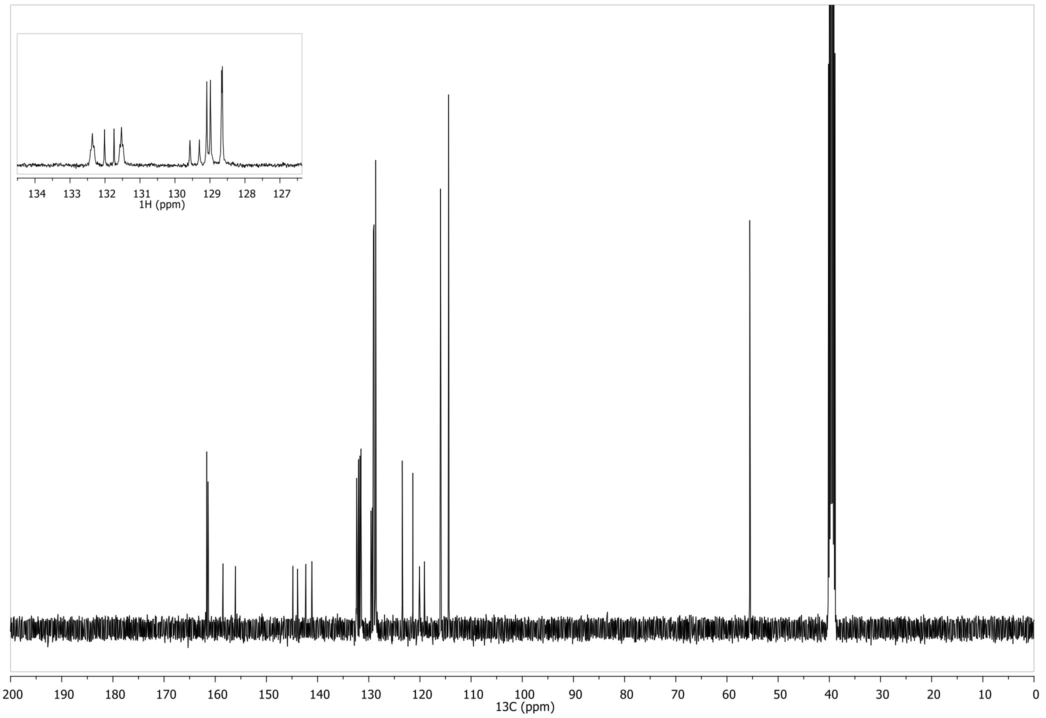

^{19}F NMR (376 MHz, DMSO-d^6) of **2**

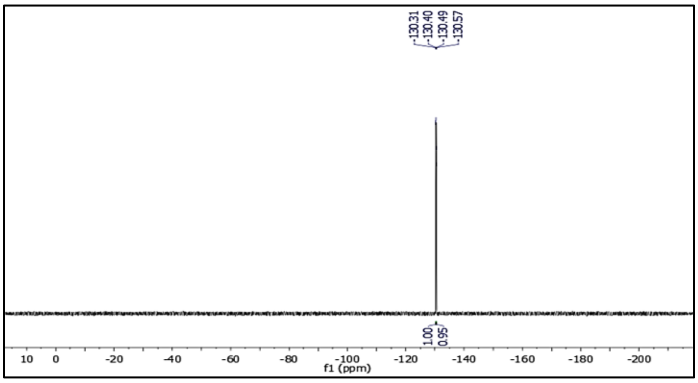

HRMS (ESI$^-$) of **2**

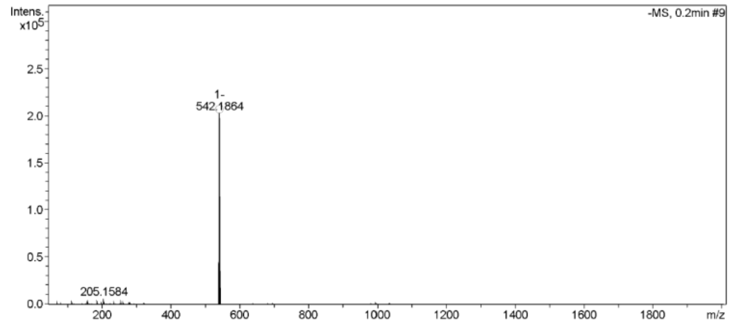

Meas. m/z	#	Ion Formula	m/z	err [mDa]	err [ppm]	rdb	N-Rule	e⁻ Conf	mSigma
542.186414	1	C33H23BF2N3O2	542.186264	-0.2	-0.3	23.5	ok	even	10.9

Figure S10: Analytical spectra for **3**

^{1}H NMR (400 MHz, $CDCl_3$)

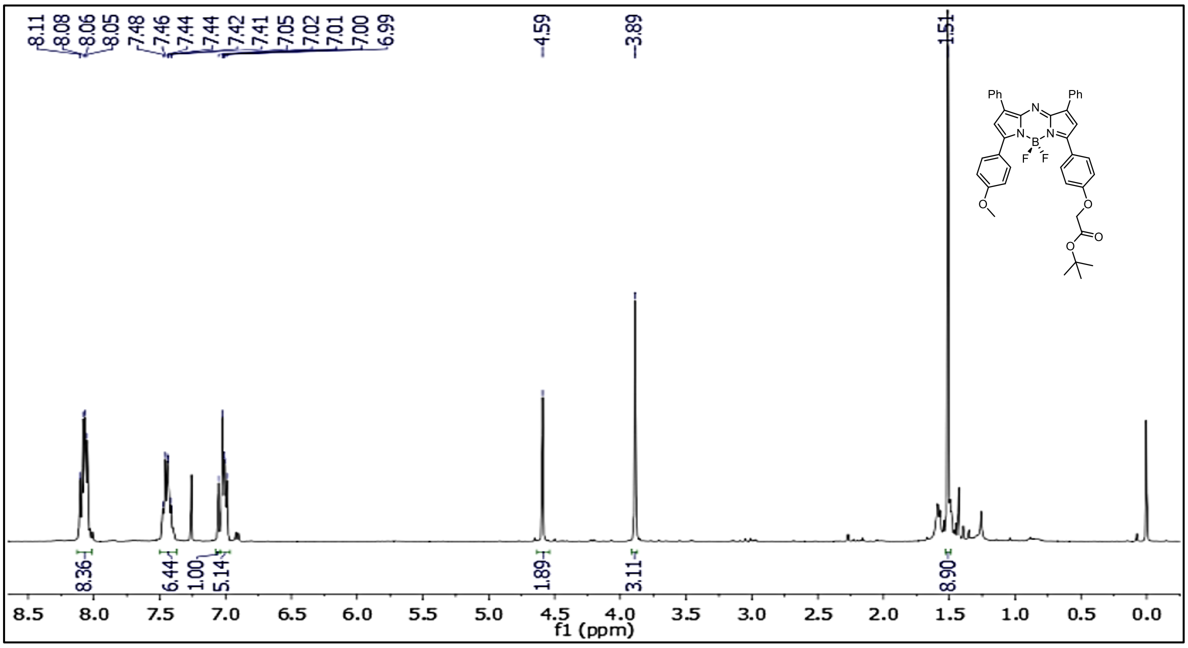

^{13}C NMR (101 MHz, $CDCl_3$)

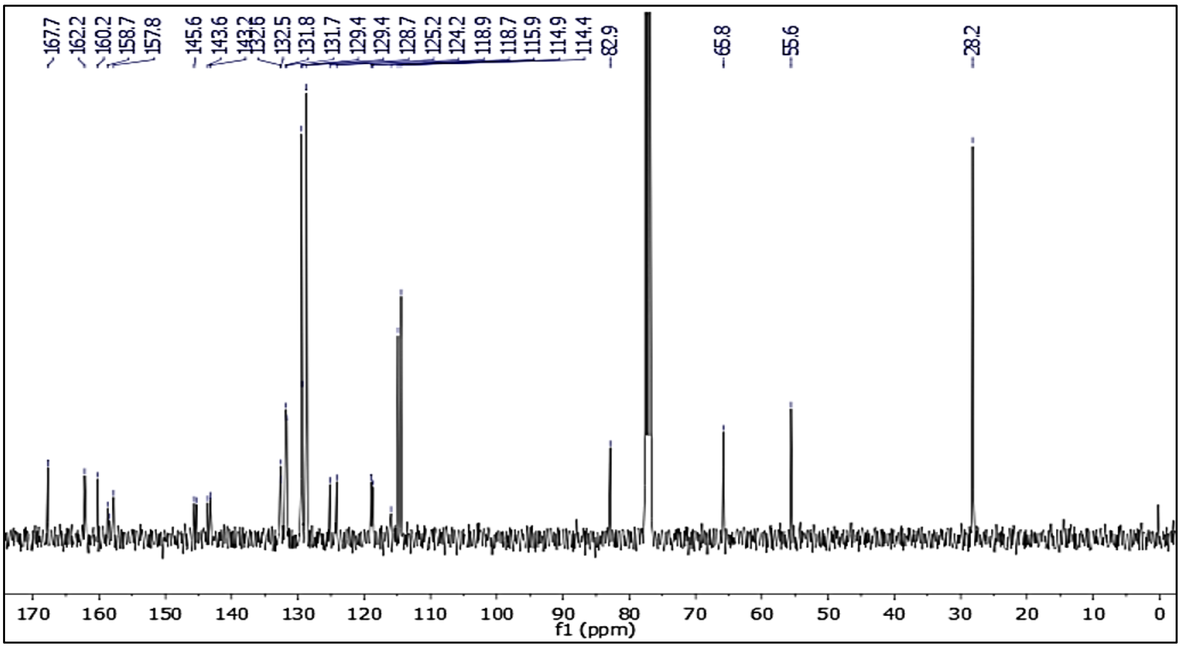

^{19}F NMR (376 MHz, $CDCl_3$)

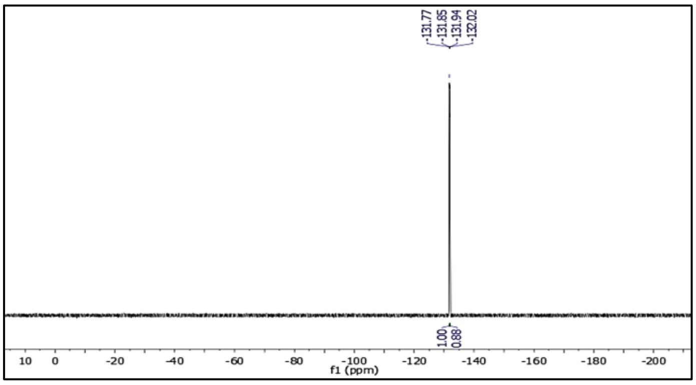

HRMS (ESI^+) of **3**

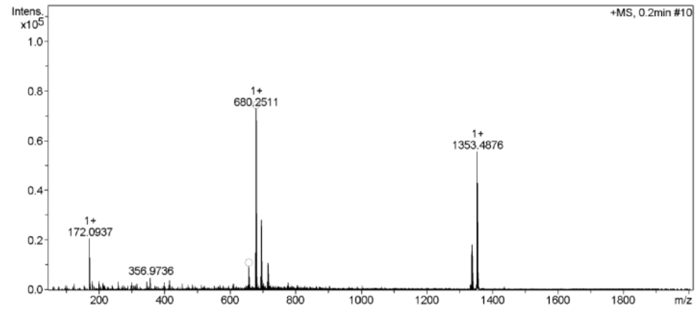

Meas. m/z	#	Ion Formula	m/z	err [mDa]	err [ppm]	rdb	N-Rule	e⁻ Conf	mSigma
658.268348	1	C39H35BF2N3O4	658.268993	-0.6	-1.0	23.5	ok	even	20.3
680.251091	1	C39H34BF2N3NaO4	680.250937	0.2	0.2	23.5	ok	even	11.9

Figure S11: Analytical spectra for **3a**

^{1}H NMR (400 MHz, DMSO-d^{6})

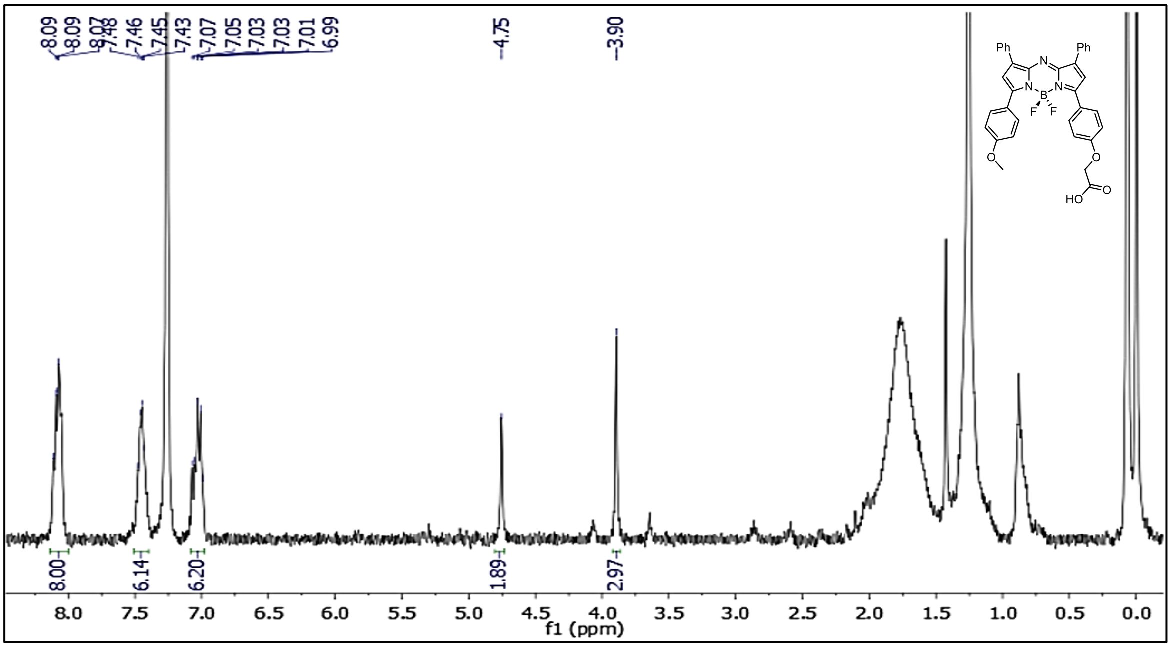

^{19}F NMR (376 MHz, $CDCl_3$)

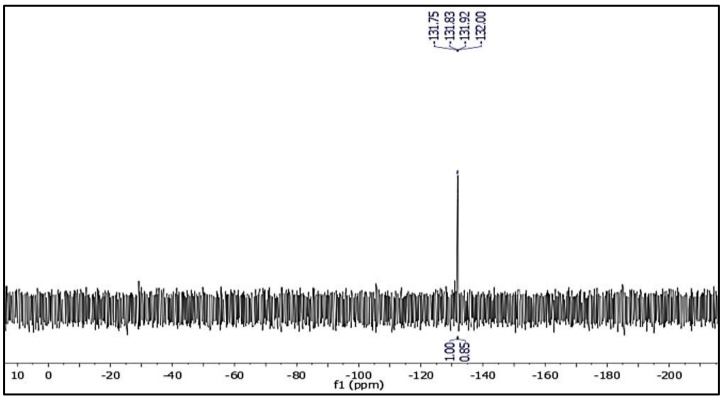

HRMS (ESI^{-}) for **3a**

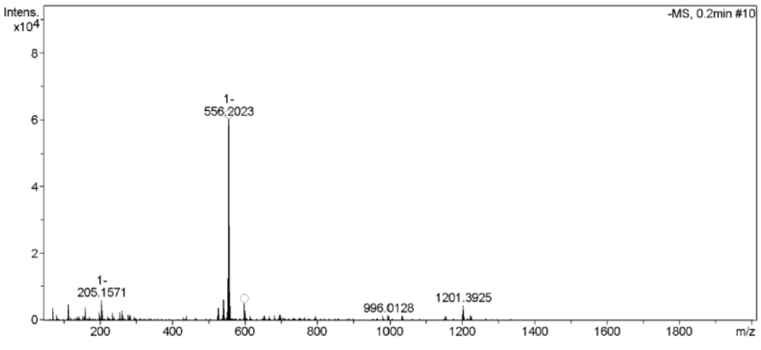

Meas. m/z	#	Ion Formula	m/z	err [mDa]	err [ppm]	rdb	N-Rule	e⁻ Conf	mSigma
556.202349	1	C34H25BF2N3O2	556.201930	0.4	0.8	23.5	ok	even	36.9
600.192938	1	C35H25BF2N3O4	600.191776	1.2	1.9	24.5	ok	even	31.1

Figure S12: Analytical spectra for **4**

^{1}H NMR (400 MHz, $CDCl_3$)

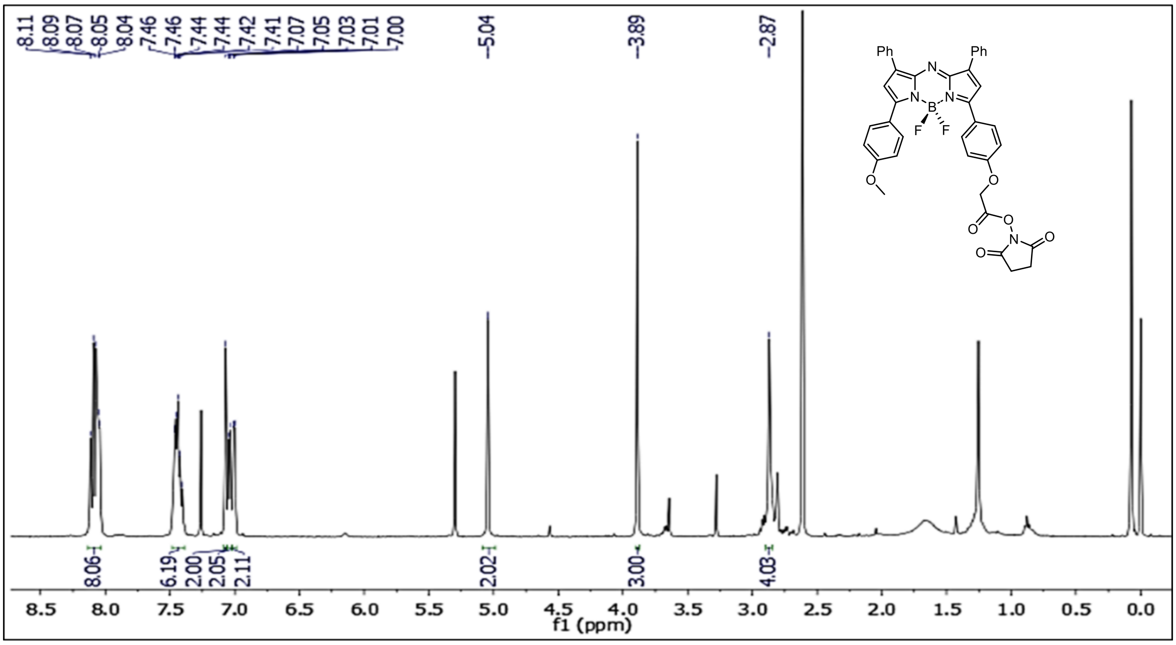

^{13}C NMR (101 MHz, $CDCl_3$)

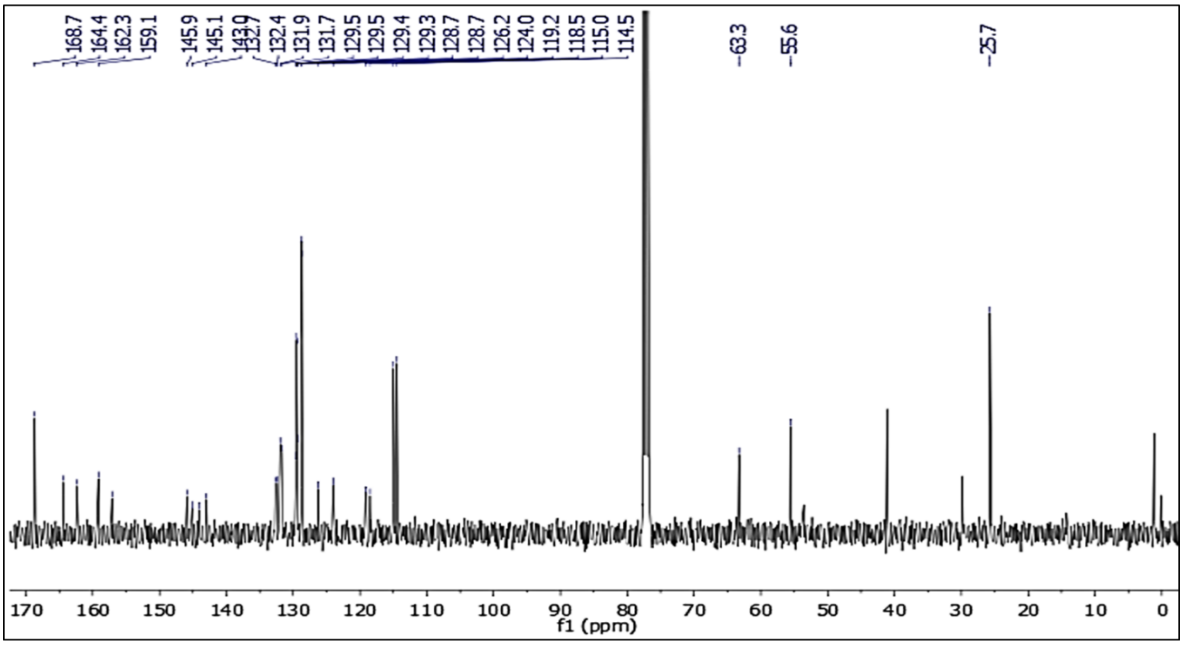

^{19}F NMR (376 MHz, $CDCl_3$)

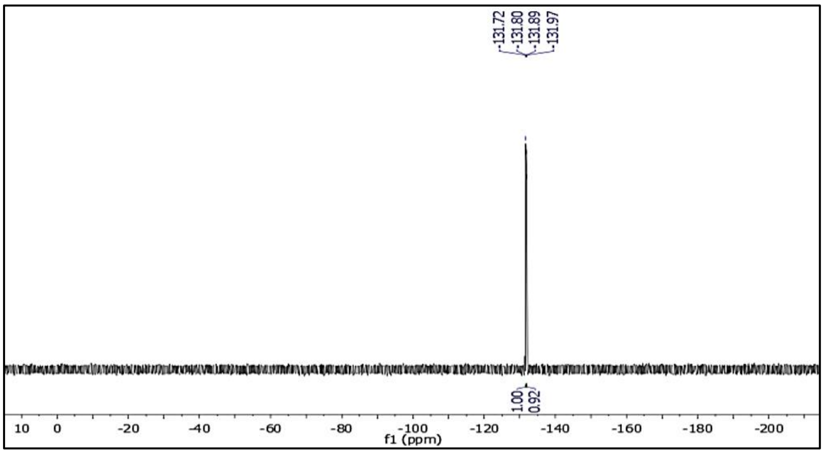

HRMS (ESI^+) for **4**

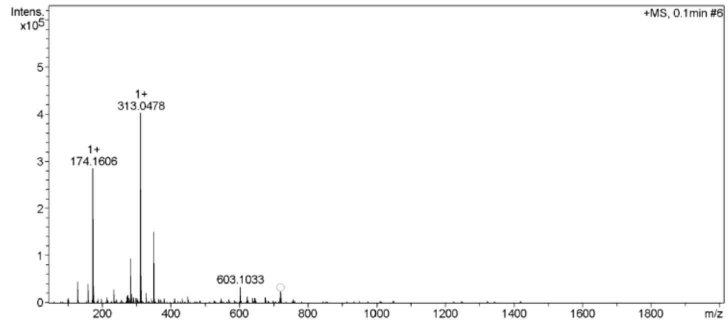

Meas. m/z	#	Ion Formula	m/z	err [mDa]	err [ppm]	rdb	N-Rule	e⁻ Conf	mSigma
721.204812	1	C39H29BF2N4NaO6	721.204715	-0.1	-0.1	26.5	ok	even	8.5

Figure S13: Analytical spectra for **7**

^{1}H NMR (400 MHz, $CDCl_3$)

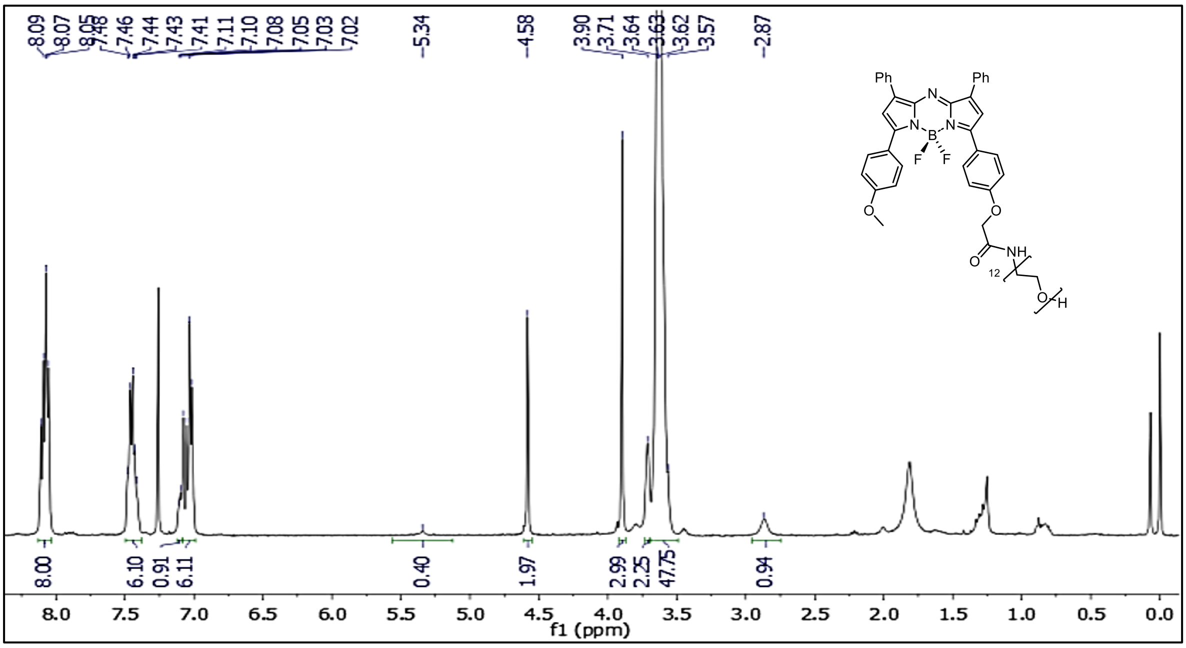

^{19}F NMR (376 MHz, $CDCl_3$)

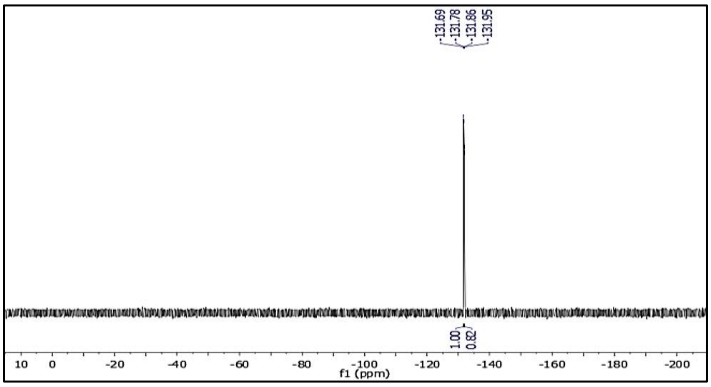

MALDI (Q-TOF) for **7**

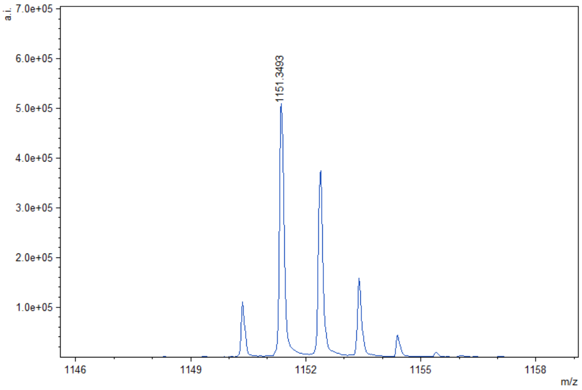

Figure S14: Analytical spectra for **8**

^{1}H NMR (400 MHz, $CDCl_3$)

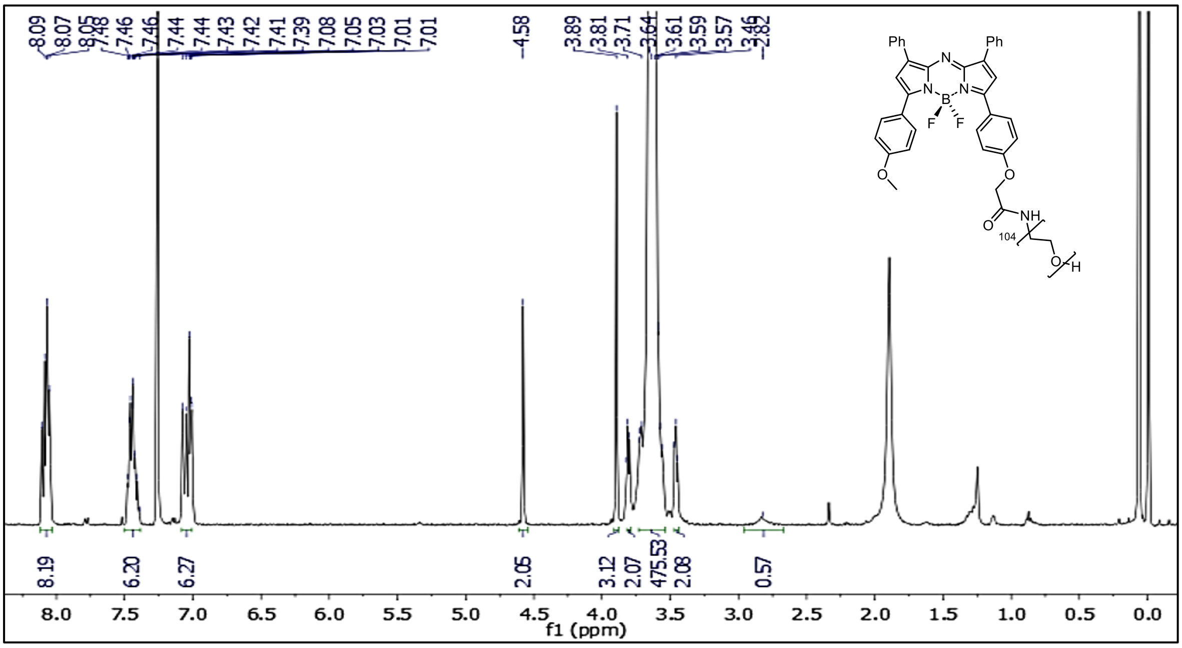

^{1}H NMR (400 MHz, $CDCl_3$)

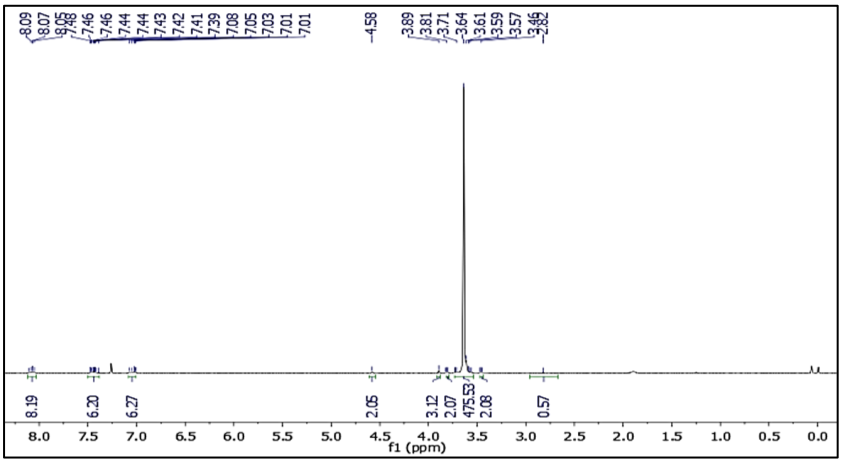

^{19}F NMR (376 MHz, $CDCl_3$)

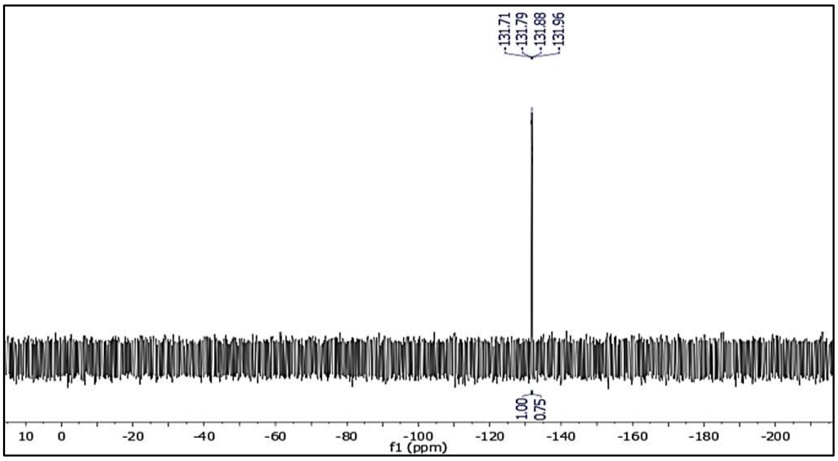

MALDI (Q-TOF) for **8**

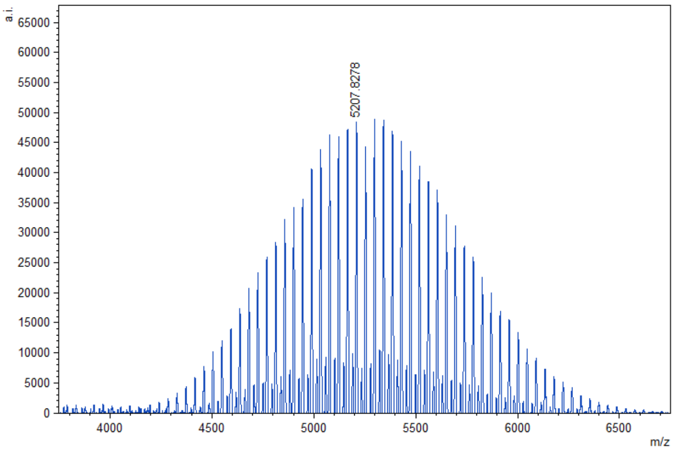

Figure S15: Analytical spectra for **9**

^{1}H NMR (400 MHz, $CDCl_3$)

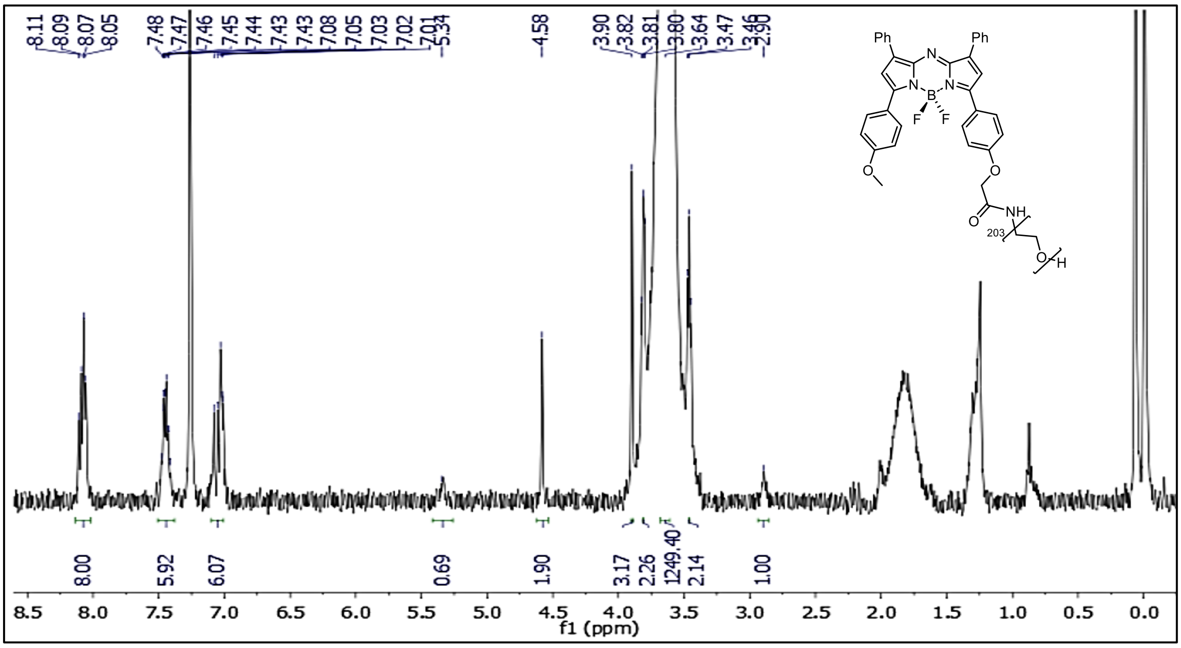

^{1}H NMR (400 MHz, $CDCl_3$)

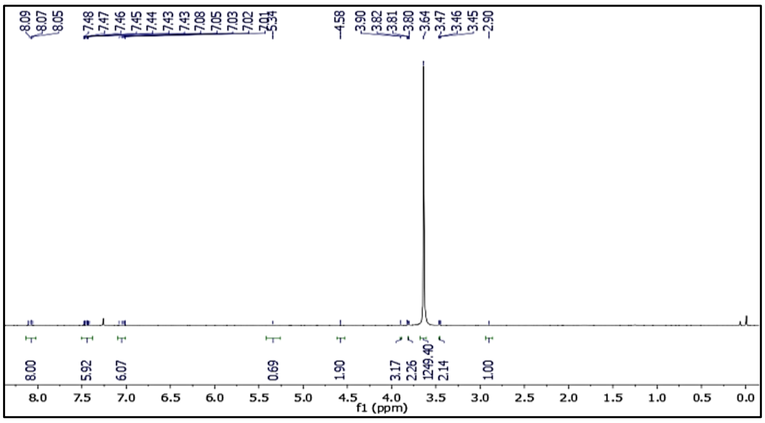

^{19}F NMR (376 MHz, $CDCl_3$)

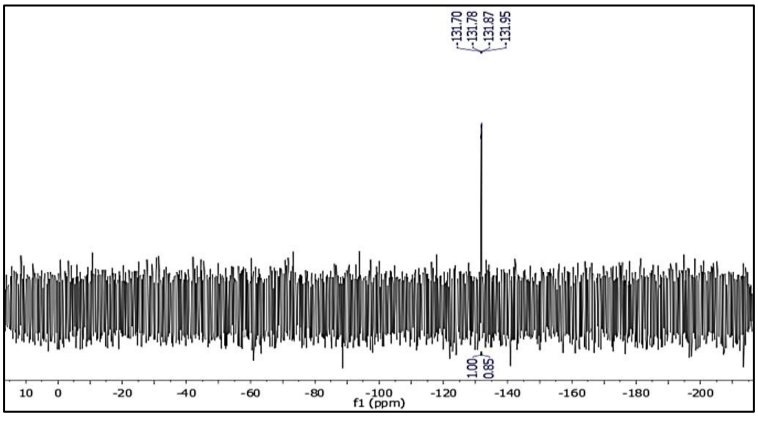

MALDI (Q-TOF) for **9**

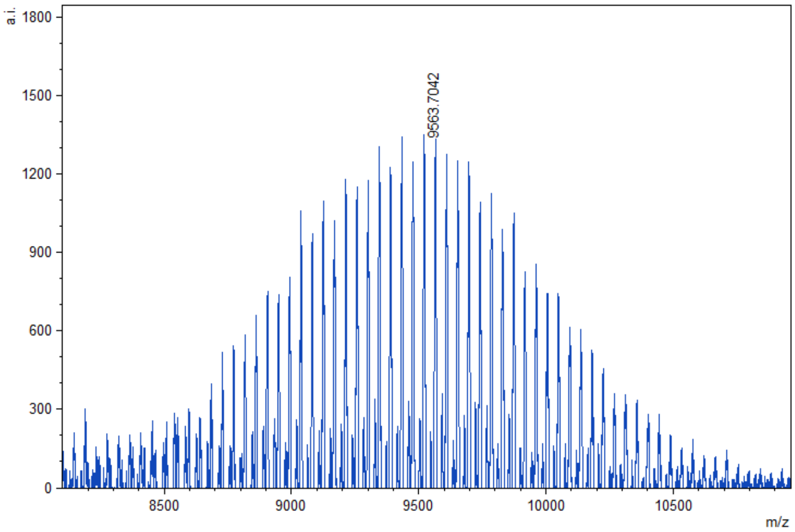

Figure S16: Analytical spectra for **10**

^{1}H NMR (400 MHz, $CDCl_3$)

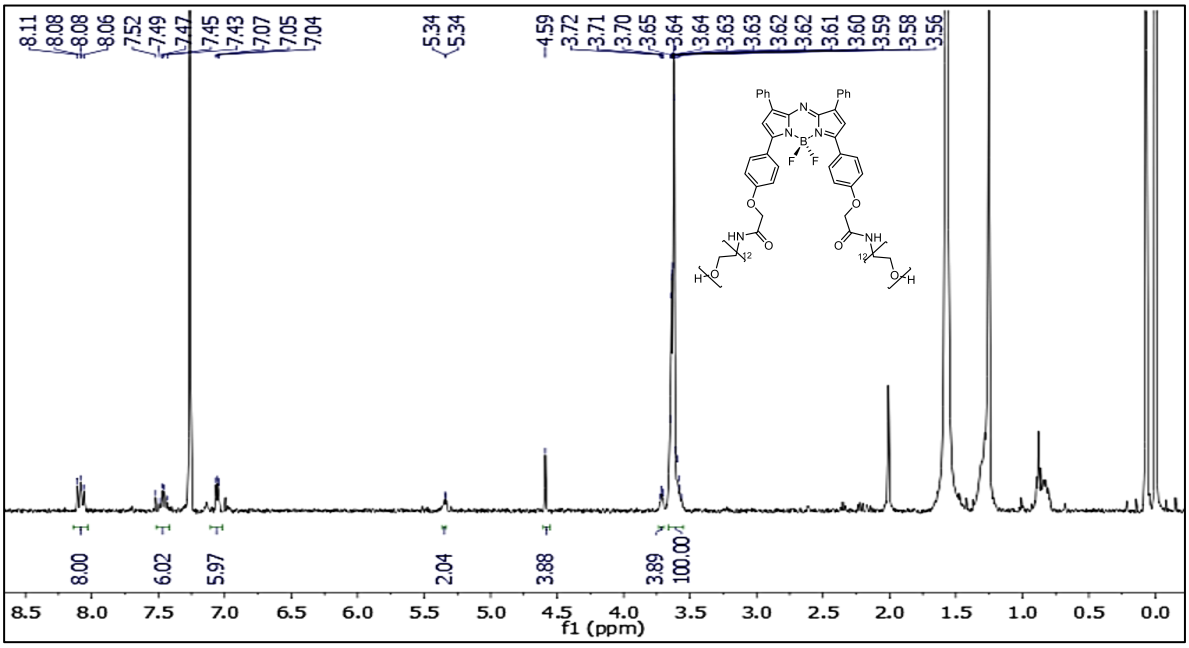

^{1}H NMR (400 MHz, $CDCl_3$)

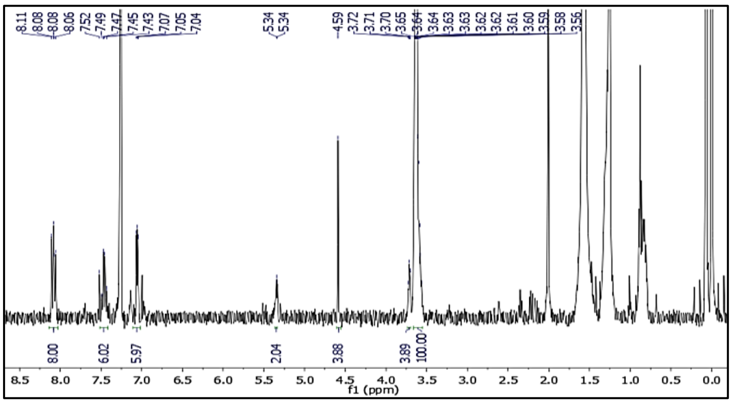

^{19}F NMR (376 MHz, $CDCl_3$)

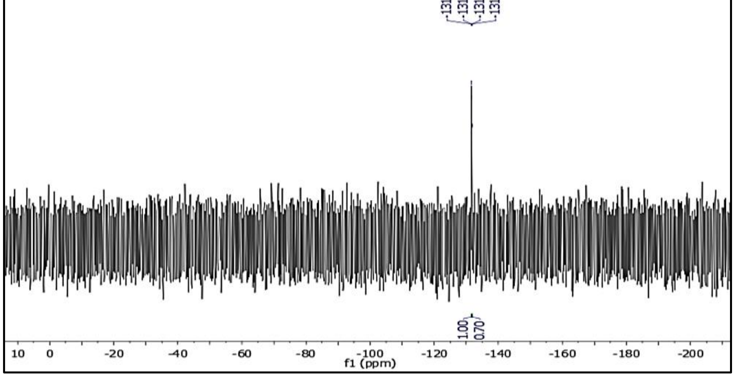

MALDI (Q-TOF) for **10**

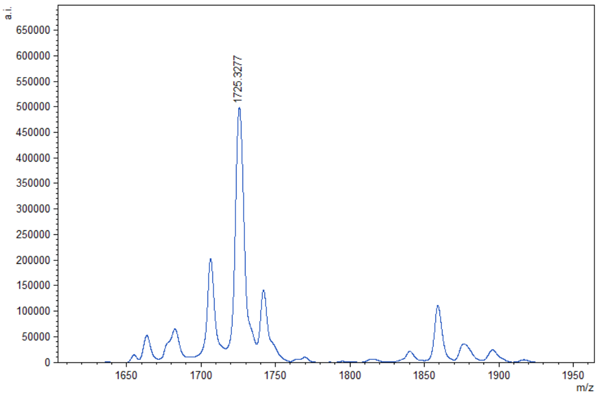

Figure S17: Analytical spectra for**11**

^{1}H NMR (400 MHz, $CDCl_3$)

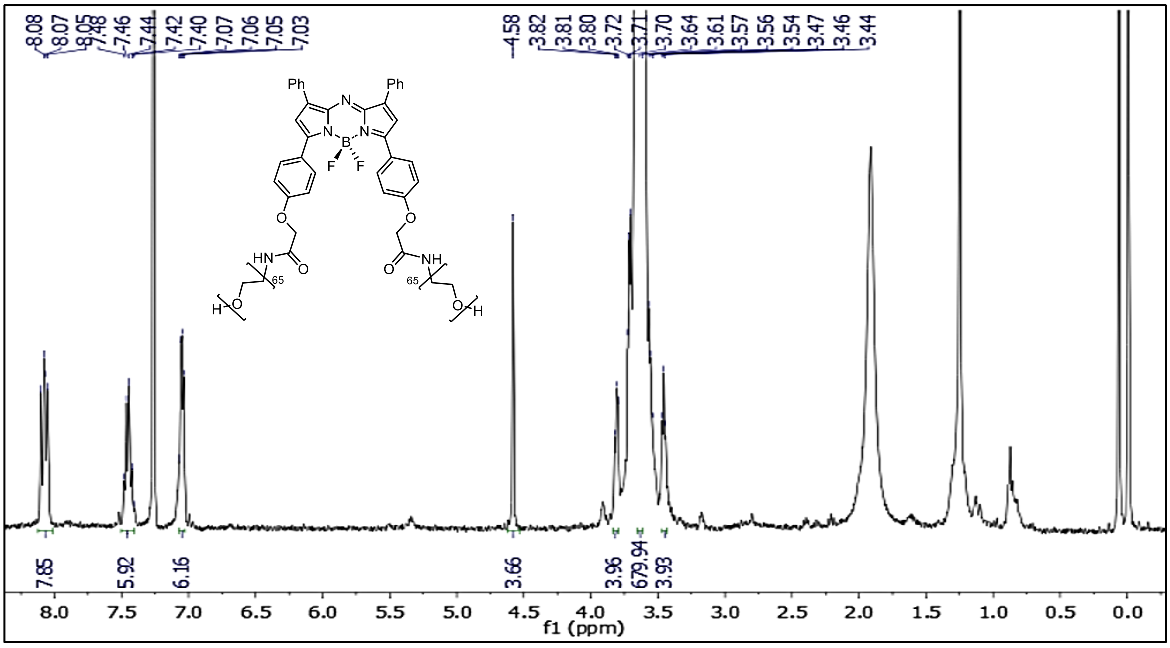

^{1}H NMR (400 MHz, $CDCl_3$)

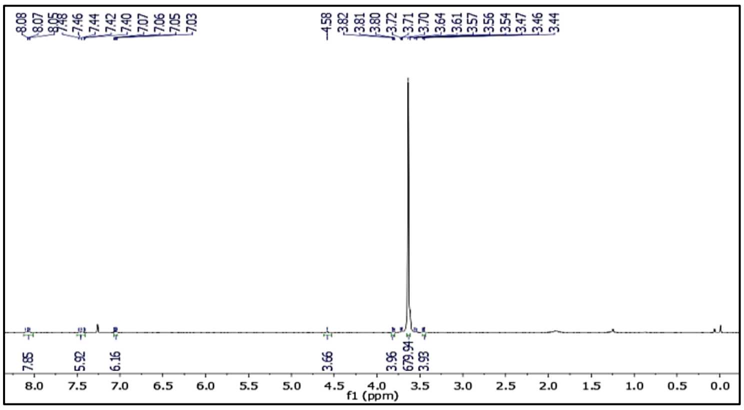

^{19}F NMR (376 MHz, $CDCl_3$)

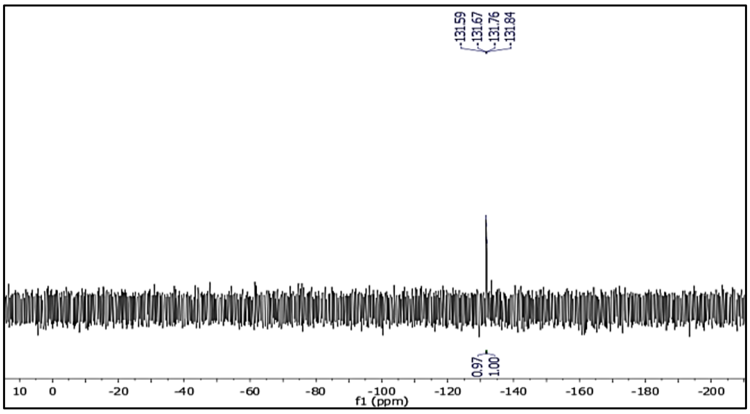

MALDI (Q-TOF) for **11**

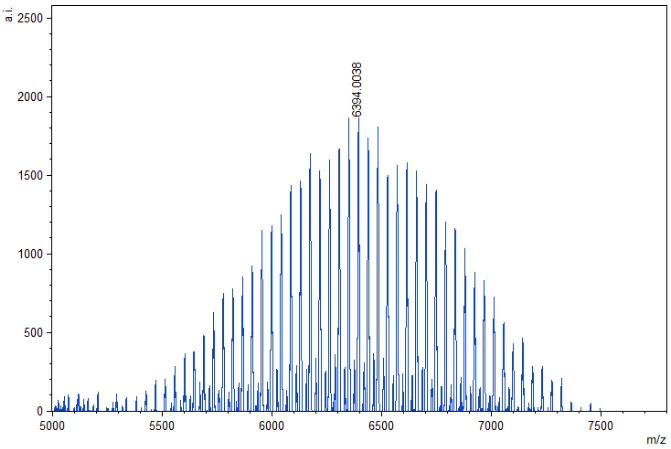

Figure S18: Analytical spectra for**12**

^{1}H NMR (400 MHz, $CDCl_3$)

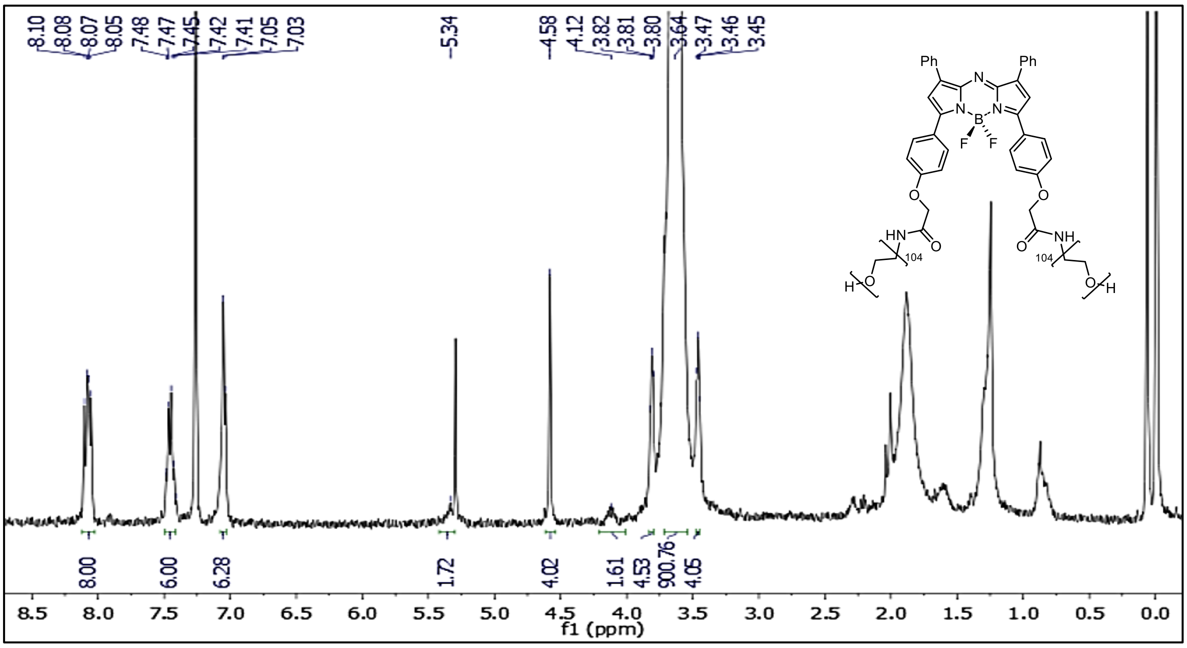

^{1}H NMR (400 MHz, $CDCl_3$)

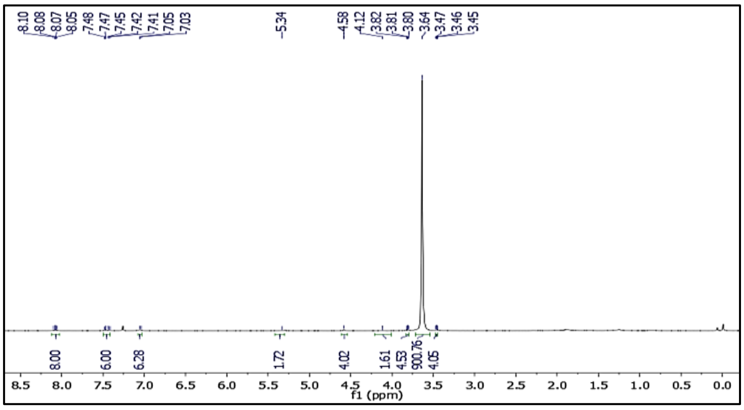

^{19}F NMR (376 MHz, $CDCl_3$)

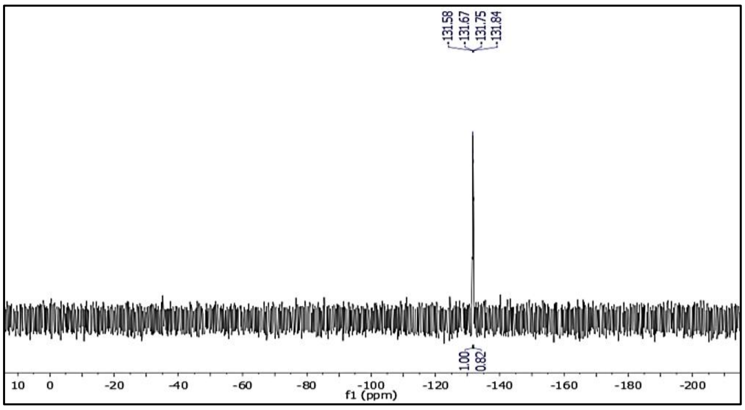

MALDI (Q-TOF) of **12**

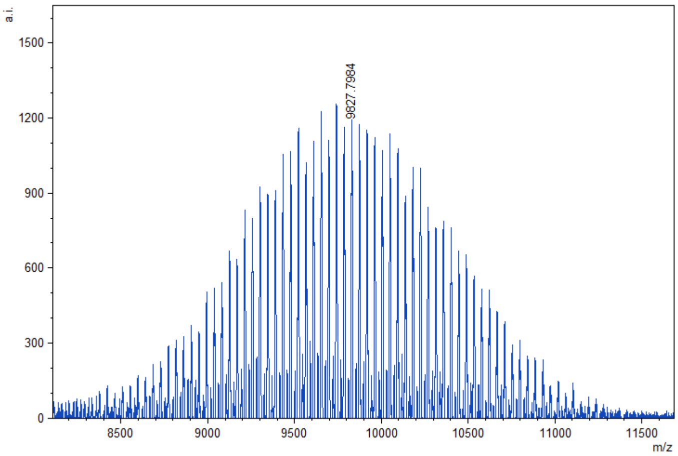

Figure S19: Analytical HPLC traces of **3, 3a, 4** and **7** – **12**.

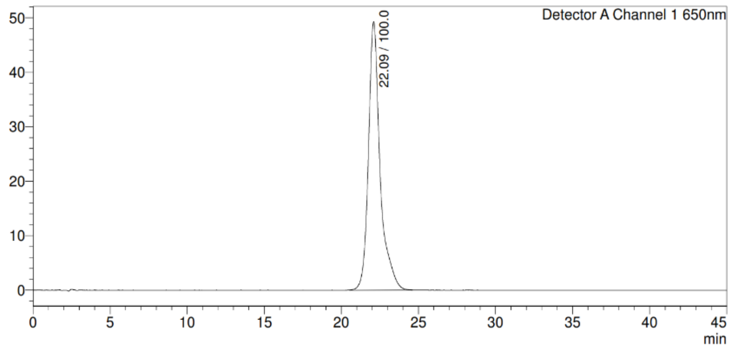

HPLC Trace of **3**. Conditions: Reverse phase-HPLC with YMC triart phenyl column and size: 150 × 4.6 mm I.D., particle size: S-5µm, 12 nm hole, detection method: UV-Vis and wavelength for detection: 650 nm and 600 nm. Eluent ACN:H_2O = 70:30 with a flow rate at 1 mL/min.

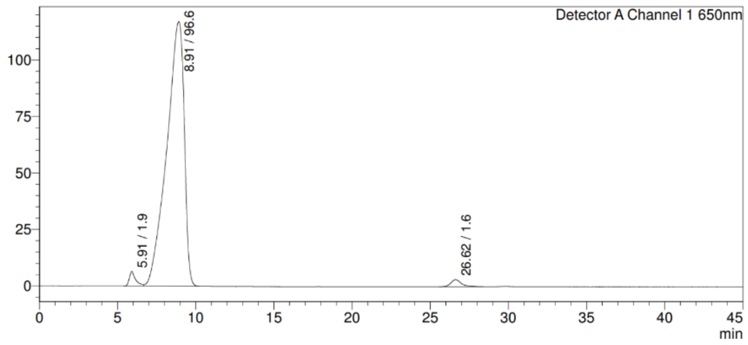

HPLC Trace of **3a**. Conditions: As above for **3**. Elution gradient of ACN:H_2O (40:60 going to 70:30) with a flow rate at 1 mL/min.

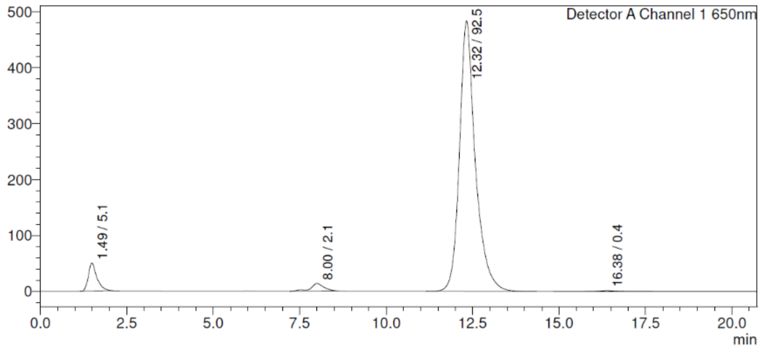

HPLC Trace of **4**. Conditions: As above for **3**. Eluent ACN:H_2O = 70:30 with a flow rate at 1 mL/min.

Mono-*O*-PEG(1.1kDa) 7

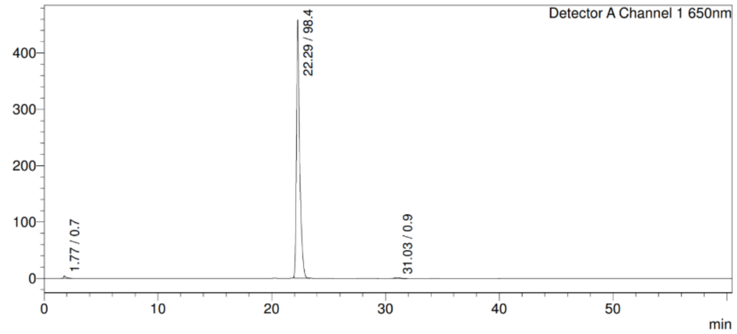

HPLC Trace of **7**. Conditions: As above for **3**. Elution gradient of ACN:H_2O (50:50 going to 70:30) with a flow rate at 1 mL/min.

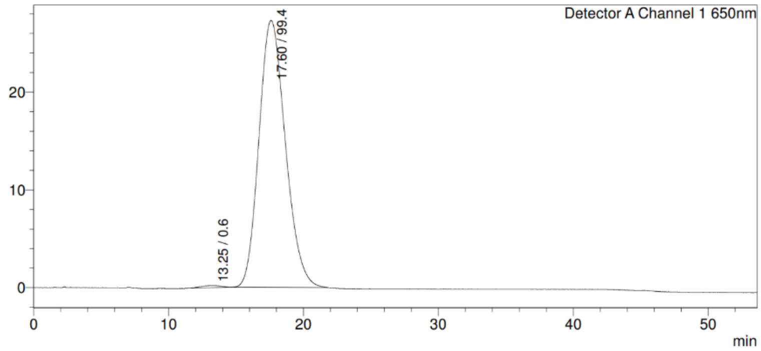

FHPLC Trace of **8**. Conditions: As above for **3**. Elution gradient of ACN:H_2O (47.5:52.5 going to 70:30) with a flow rate at 1 mL/min.

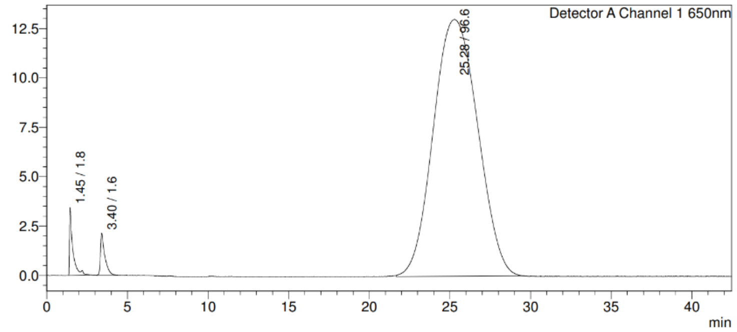

HPLC Trace of **9**. Conditions: As above for **3**. Elution gradient of ACN:H_2O (40:60 going to 70:30) with a flow rate at 1 mL/min.

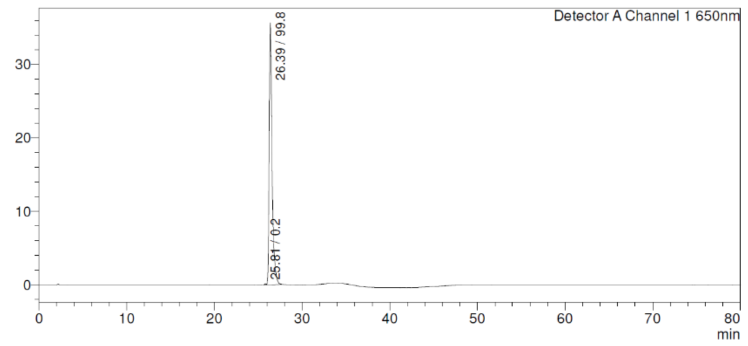

HPLC Trace of **10**. Conditions: As above for **3**. Elution gradient of ACN:H_2O (42.5:57.5 going to 70:30) with a flow rate at 1 mL/min.

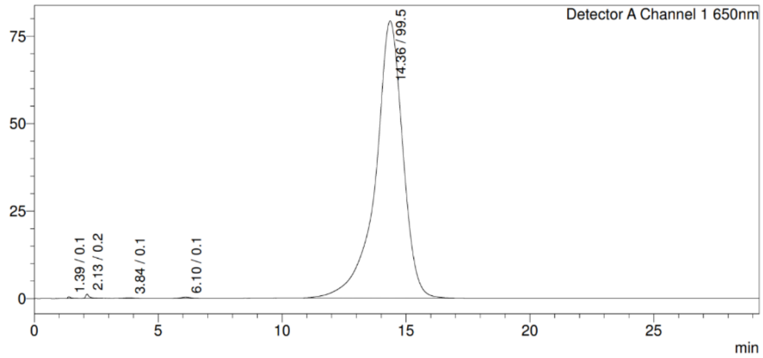

HPLC Trace of **11**. Conditions: As for **3** above. Elution gradient of ACN:H_2O (45:55 going to 70:30) with a flow rate at 1 mL/min.

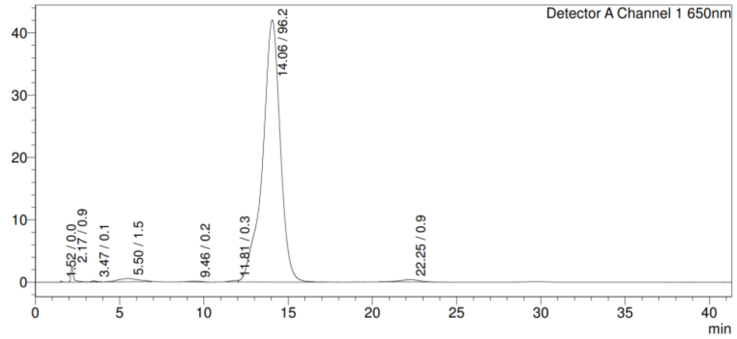

HPLC Trace of **12**. Conditions: As above for **3**. Elution gradient of ACN:H_2O (45:55 going to 70:30) with a flow rate at 1 mL/min.

References

1. Murtagh, J.; Frimannsson, D.O.; O'Shea, D.F. Azide conjugatable and pH responsive near-infrared fluorescent imaging probes. *Org. Lett.*, **2009**, 11, 5386–5389.

2. Gorman, A.; Killoran, J.; O'Shea, C.; Kenny, T.; Gallagher, W.M.; O'Shea, D.F. In vitro demonstration of the heavy-atom effect for photodynamic therapy. *J. Am. Chem. Soc.*, **2004**, 126, 10619-10631.
